# Implications of genetic variation of common Drug Metabolizing Enzymes and ABC Transporters among the Pakistani Population

**DOI:** 10.1038/s41598-019-43736-z

**Published:** 2019-05-13

**Authors:** Nasir Ali Afsar, Henrike Bruckmueller, Anneke Nina Werk, Muhammad Kashif Nisar, H. R. Ahmad, Ingolf Cascorbi

**Affiliations:** 1 Jinnah Medical and Dental College, Sohail University, 22-23 Shaheed-e-Millat Road, Karachi, 75400 Pakistan; 2 Institute of Experimental and Clinical Pharmacology, Christian Albrechts University Kiel, Hospitalstr. 4, Kiel, 24105 Germany; 3 Department of Internal Medicine I, University Medical Center Hamburg-Eppendorf, Hamburg, Germany; 4 Department of Biological and Biomedical Sciences, The Aga Khan University, Karachi, Pakistan; 5 Sindh Institute of Urology and Transplantation, Karachi, Pakistan; 6 Present Address: Liaquat National Hospital & Medical College, Karachi, Pakistan

**Keywords:** Rare variants, Translational research

## Abstract

Genetic polymorphism of drug metabolizing enzymes and transporters may influence drug response. The frequency varies substantially between ethnicities thus having implications on appropriate selection and dosage of various drugs in different populations. The distribution of genetic polymorphisms in healthy Pakistanis has so far not been described. In this study, 155 healthy adults (98 females) were included from all districts of Karachi. DNA was extracted from saliva and genotyped for relevant SNVs in *CYP1A1, CYP2B6, CYP2C9, CYP2C19, CYP2D6, CYP3A4* and *CYP3A5* as well as *ALDH3A1, GSTA1, ABCB1 and ABCC2*. About 64% of the participants were born to parents who were unrelated to each other. There was generally a higher prevalence (p < 0.05) of variant alleles of CYP450 1A2, 2B6, 2C19, 3A5, ALDH3A1, GSTM1 as well as ABCB1 and ABCC2 in this study cohort than in other ethnicities reported in the HapMap database. In contrast, the prevalence of variant alleles was lower in *GSTA1*. Therefore, in the Pakistani population sample from Karachi a significantly different prevalence of variant drug metabolizing enzymes and ABC transporters was observed as compared to other ethnicities, which could have putative clinical consequences on drug efficacy and safety.

## Introduction

The concept of choosing the right medicine for right person is not new. However, pharmacogenomic research has enabled us to predict an adverse outcome of administering a medication that would formerly have been judged to be generally safe and effective^1^. Due the initiative of the Clinical Pharmacogenetics Implementation Network and others^2^, many drugs in the USA are now dispensed with FDA advised pharmacogenetic warning labels. A detailed list of pharmacogenetic markers is available online at the FDA website (www.fda.gov/Drugs/ScienceResearch/ucm572698.htm). Drug regulatory agencies like the European EMA are following the lead. However, such data stems mainly from the West, which may not be applicable to other parts of the world.

Genetic variability of drug metabolizing enzymes and drug transporters has been associated with interindividual differences in pharmacokinetics and pharmacodynamics. Such differences may result in variation in drug efficacy, safety and treatment outcomes in a number of frequently prescribed drugs^3^. A notable example is that of pharmacogenetic peculiarities of Ashkenazi Jewish population who are reported to have important therapeutic implications, such as *VKORC1* gene polymorphism necessitating warfarin dose adjustment^4^. Hence, interindividual genetic differences within but also between various ethnic groups are considered to be an important contributory factor to the variability of drug responses^5^. In this study, we characterized single nucleotide variants (SNVs) of select phase I enzymes (CYPs and ALDHs), phase II enzymes (GSTs, UGTs, TPMPTs and NATs) and transporters involved in drug metabolism in a population of 155 Karachiites in Pakistan, because no such studies are reported for this population. Further, we compared the variant allele frequency with allele frequencies reported for major ethnic groups in HapMap database and reported the differences between our population and each of those representative groups in HapMap.

It is estimated that 75–80% of prescribed drugs are metabolized by oxidizing phase I cytochrome P450 enzymes such as CYP3A4 and 5, CYP2D6, CYP2C19 or CYP2C9, with CYP3A4/5 metabolizing more than half of currently prescribed drugs^6^. In addition, phase II enzymes catalyze the conjugation of xenobiotic metabolites with various hydrophilic molecules to render them less toxic and more polar, thus favouring their excretion from the body. Such reactions are catalyzed by different enzyme groups, such as GSTs (glutathione S-transferases), UGTs (UDP-glucuronosyl transferases), TPMTs (thiopurine S-methyl transferases), NATs (N-acetyl transferases)^7^. GSTs also support detoxification reactions^8^ and play an important role in preventing oxidative stress^9^.

Additionally, ALDHs (aldehyde dehydrogenases) are phase I xenobiotic metabolizing enzymes which have diverse functions, such as neutralization of toxic aldehydes during lipid peroxidation^10^, coenzyme Q synthesis^11^, prevention of tobacco smoke-induced respiratory epithelial cytotoxicity^12^, metabolism of cyclophosphamide^8^ and ethanol^13^ among other functions. Thus, altered ALDH function could predispose individuals to numerous medical conditions such as atherosclerosis, dementia, infertility, cancers. This becomes further complicated if the biological role shows a gene-dose effect, as we previously reported for ALDH3A1^8^.

ATP binding cassette (ABC) transporters are a group of membrane transporters, which transport many xenobiotics, including drugs, in and out of various cells. In fact, some of them were called multi-drug resistance proteins because of this role. Of notable interest are ABCB1 and ABCC2 transporters. Their substrates include many drugs, including anticancer drugs, HIV protease inhibitors, antibiotics, beta blockers, statins, anticonvulsants, opiates^14^.

As outlined above, ethnic differences exist in the prevalence of genetic variants of the enzymes and transporters^15^. Hence, genetic characterization of patients may prove valuable in predicting therapeutic outcomes^16–18^. We reported previously^19^ that significant differences exist in the frequencies of polymorphic genes involved in metabolism and cellular transport of breast cancer chemotherapy among breast cancer patients from Karachi, as compared to some ethnic groups reported in HapMap database (https://www.ncbi.nlm.nih.gov/snp). However, that study was limited because of the small sample size used and the absence of data from a healthy population.

To the best of our knowledge, there is no comprehensive study on this topic involving South Asia and adjoining regions. Hence, we designed this study to explore the genotype profiles among healthy adults from different ethnicities living in Karachi, allowing us to compare them with those reported for other major ethnic groups in the HapMap database.

## Results

Table 1 shows the baseline characteristics of the study population. A total of 155 healthy Pakistani adults (98 females and 57 males) with a median age of 19 years (range: 18–70 years) were included in this study. Participants were from all districts of Karachi and belonged to various major ethnic groups within Pakistan. Ethnicity was classified according to their mother tongue, including Balochi, Gujrati, Pashtun, Punjabi, Seraiki, Sindhi, and other minor groups. As expected, the local Urdu-speaking community with heterogeneous Indian ancestry, collectively described as *Muhajir* (*Arabic/Urdu*; immigrants) featured most in our population. Since consanguineous marriages are common in Pakistan^20^, we sought information regarding this fact. Most individuals declared that their parents were not related to each other. Some of the participants, labelled as ‘mixed lineage’ had grandparents from different ethnicities.

**Table 1:** Baseline characteristics of study participants.

Participant Characteristics		Participant		Mother		Father	
		N	%	n	%	n	%
Gender	Female:	98	63.2				
	Male:	57	36.8				
Ethnicity	Urdu Speaking:	92	59.4	101	65.2	96	61.9
	Sindhi:	10	6.5	11	7.1	12	7.7
	Gujrati:	9	5.8	11	7.1	13	8.4
	Punjabi:	8	5.2	11	7.1	11	7.1
	Pashtun:	5	3.2	6	3.9	5	3.2
	Seraiki:	2	1.3	2	1.3	2	1.3
	Others:	6	3.9	6	3.9	7	4.5
	Mixed lineage:	18	11.6				
	Not declared	5	3.2	7	4.5	9	5.8
Consanguinity	No:	99	63.9				
	Yes:	55	35.5				
District	East:	71	45.8				
	Central:	33	21.3				
	South:	28	18.1				
	Korangi:	9	5.8				
	Malir:	8	5.2				
	West:	3	1.9				

Table 2 shows the frequency distribution of SNVs and genotypes. Genotypes were in Hardy-Weinberg equilibrium. Some of the samples could not be genotyped completely, apparently due to low DNA quantity or quality. Haplotype and diplotype analyses were carried out where applicable. Table 2 shows that in our population the percent frequency of wild type genotype was as follows:

- **Phase I enzymes:** CYP1A1 42% (heterozygous 46%; homozygous variant 12%), CYP2B6 20% (heterozygous 54%; homozygous variant 26%), CYP2C9 71% (heterozygous 26%), CYP2C19 27% (heterozygous 48%; homozygous variant 25%), CYP2D6 extensive metabolizers 74% (25% intermediate metabolizers, 1% poor metabolizers), CYP3A4 98% (heterozygous 2%), CYP3A5 1% (heterozygous 38%, homozygous variant 61%), ALDH3A1 8% (heterozygous 50%, homozygous variant 42%).
- **Phase II enzymes:** GSTA1 49% (heterozygous 41%, homozygous variant 10%), GSTM1 null 59%.
- **ABC Transporters:** ABCB1 wildtype haplotype 10%, ABCC2 wildtype haplotype 50%

**Table 2:** Allele and diplotype frequencies of SNVs in drug metabolizing enzymes and ABC transporters (n = 155 healthy adults).

Gene	rs number	SNV	Genotyping Success (%)	Minor allele (%)	Genotype/Diplotype	Participants		95% Confidence Interval		
						(n)	(%)			
***CYP1A1***	rs1799814	g.2452C > A	92.3	1.7	*1/*1	61	42.4	34.29	—	50.43
	rs1048943	g.2454A > G	96.1	11.1	*1/*2A	42	29.2	21.74	—	36.59
	rs4646903	g.3798 T > C	92.9	32.6	*1/*2B	19	13.2	7.67	—	18.72
					*1/*2C	1	0.7	0.00	—	2.05
					*1/*4	4	2.8	0.09	—	5.46
					*2A/*2A	6	4.2	0.90	—	7.43
					*2A/*2B	7	4.9	1.35	—	8.37
					*2A/*4	1	0.7	0.00	—	2.05
					*2B/*2B	3	2.1	0.00	—	4.42
***CYP2B6***	rs3745274	g.516G > T	94.8	36.1	*1/*1	29	20.4	13.79	—	27.05
	rs2279343	g.785A > G	80.0	48.0	*1/*4	10	7.0	2.83	—	11.25
	rs3211371	g.1459C > T	79.4	8.1	*1/*5	12	8.5	3.88	—	13.03
					*1/*6	51	35.9	28.02	—	43.81
					*1/*9	3	2.1	0.00	—	4.48
					*4/*4	4	2.8	0.10	—	5.54
					*4/*6	8	5.6	1.84	—	9.43
					*4/*7	1	0.7	0.00	—	2.08
					*4/*33	2	1.4	0.00	—	3.35
					*5/*6	4	2.8	0.10	—	5.54
					*6/*6	17	12.0	6.63	—	17.31
					*6/*7	*1*	0.7	−0.67	—	2.08
***CYP2C9***	rs1799853	c.430C > T	94.8	6.8	*1/*1	108	70.6	63.37	—	77.81
	rs1057910	c.1075A > C	97.4	9.9	*1/*2	16	10.5	5.61	—	15.31
					*1/*3	24	15.7	9.92	—	21.45
					*2/*3	0	0.0	—	—	—
					*2/*2	4	2.6	0.09	—	5.14
					*3/*3	1	0.7	0.00	—	1.93
***CYP2C19***	rs4244285	c.681G > A	100	31.0	*1/*1	42	27.1	20.10	—	34.09
	rs12248560	g.-806C > T	98.1	18.1	*1/*2	46	29.7	22.49	—	36.87
					*1/*17	29	18.7	12.57	—	24.85
					*2/*2	15	9.7	5.02	—	14.33
					*2/*17	20	12.9	7.63	—	18.18
					*17/*17	3	1.9	0.00	—	4.10
***CYP2D6***	rs1065852	g.100C > T	78.1	17.8	*1/*1 (*EM*)	56	73.7	63.78	—	83.58
	rs5030655	g.1707delT	49.7	0.6	*1/*10 (*IM*)	3	3.9	−0.43	—	8.33
	rs3892097	g.1846C > T	49.7	11.0	*1/*4 (*IM*)	14	18.4	9.71	—	27.14
	rs35742686	g.2549delA	96.1	0.0	*1/*6 (*IM*)	1	1.3	0.00	—	3.88
	rs5030656	g.2615-g.2617delAAG	90.3	0.4	*1/*9 (*IM*)	1	1.3	0.00	—	3.88
					*4/*10 (*PM*)	1	1.3	0.00	—	3.88
***CYP3A4***	rs35599367	g.15389C > T	100	1.0	*1/*1	152	98.1	95.91	—	100.21
					*1/*22	3	1.9	0.00	—	4.09
					*22/*22	0		-	—	—
***CYP3A5***	rs776746	g.6986G > A	78.7	20.1	*1/*1	1	0.8	0.00	—	2.40
					*1/*3	47	38.5	29.89	—	47.15
					*3/*3	74	60.7	51.99	—	69.33
***ALDH3A1***	rs2228100	c.985G > C	95.5	33.1	*C*/*C*	12	8.1	3.71	—	12.51
					*C*/*G*	74	50.0	41.94	—	58.06
					*G*/*G*	62	41.9	33.94	—	49.84
***GSTA1***	rs3957357	g.−69C > T	99.4	30.5	*A/*A (*CC*/*GG*)	75	48.7	40.81	—	56.59
	rs3957356	g.-52G > A	99.4	30.5	*A/*B (*CG*/*TA*)	64	41.6	33.78	—	49.34
					*B/*B (*TT*/*AA*)	15	9.7	5.07	—	14.41
***GSTM1***		*0 (*null*)	99.4	30.1	*1/*1, *0/*1	60	39.0	31.26	—	46.66
(*Gene Deletion*)					*0/*0	91	59.1	51.32	—	66.86
***ABCB1***	rs1128503	g. c.1236C > T	97.4	59.3	*C*/*C*	27	17.9	11.77	—	23.99
					*C*/*T*	68	45.0	37.10	—	52.96
					*T*/*T*	55	36.4	28.75	—	44.09
	rs2032582	c.2677G > T/A	96.8	T: 58.6	*G*/*G*	22	16.5	10.23	—	22.85
				A: 6.3	*G*/*T*	57	42.9	34.45	—	51.27
					*T*/*T*	54	40.6	32.25	—	48.95
					*G*/*A*	5	3.8	0.53	—	6.99
					*T*/*A*	12	9.0	4.15	—	13.89
					*A*/*A*	1	0.8	−0.72	—	2.22
	rs1045642	c.3435C > T	98.7	53.6	*C*/*C*	31	20.3	13.89	—	26.63
					*C*/*T*	80	52.3	44.38	—	60.20
					*T*/*T*	42	27.5	20.37	—	34.53
					*1/*1 (*CGC*/*CGC*)	14	9.6	4.81	—	14.37
					*1/*2 (*CGC*/*TT* (>*A*)*T*)	37	25.3	18.28	—	32.40
					*2/*2 (*TTT*/*A*)	33	22.6	15.82	—	29.38
					*Mixed*	62	42.5	34.44	—	50.48
***ABCC2***	rs717620	c.-24C > T	98.7	14.6	*C*/*C*	113	73.9	66.90	—	80.82
					*C*/*T*	37	24.2	17.39	—	30.97
					*T*/*T*	4	2.6	0.09	—	5.13
	rs2273697	c.1249G > A	97.4	24.2	*G*/*G*	84	55.6	47.71	—	63.55
					*G*/*A*	61	40.4	32.57	—	48.23
					*A*/*A*	6	4.0	0.84	—	7.10
	rs3740066	c.3972C > T	99.4	37.3	*C*/*C*	58	37.7	30.01	—	45.31
					*C*/*T*	72	46.8	38.87	—	54.63
					*T*/*T*	20	13.0	7.68	—	18.30
					*H1* (*CGC*)	146	50.0	58.11	—	41.89
					*H2* (*CAC*)	39	13.4	18.89	—	7.83
					*H9* (*CGT*)	43	14.7	20.47	—	8.99
					*H12* (*TGT*)	31	10.6	15.61	—	5.63
					*H13* (*CAT*)	23	7.9	12.26	—	3.50
					*H14* (*TAC*)	9	3.1	5.89	—	0.27
					*H15* (*TAT*)	1	0.3	1.28	—	−0.60
					*H1*/*H1*	22	15.1	20.88	—	9.26
					*H1*/*H2*	32	21.9	28.63	—	15.21
					*H1*/*H9*	28	19.2	25.57	—	12.79
					*H1*/*12*	16	11.0	16.04	—	5.88
					*H1*/*13*	17	11.6	16.83	—	6.45
					*H1*/*14*	9	6.2	10.07	—	2.25
					*H2*/*H2*	3	2.1	4.38	—	−0.28
					*H2*/*H13*	1	0.7	2.03	—	−0.67
					*H9*/*H9*	3	2.1	4.38	—	−0.28
					*H9*/*H12*	7	4.8	8.26	—	1.32
					*H9*/*H13*	1	0.7	2.03	—	−0.67
					*H9*/*H15*	1	0.7	2.03	—	−0.67
					*H12*/*H12*	4	2.7	5.37	—	0.11
					*H13*/*H13*	2	1.4	3.28	—	−0.54

**Figure 1:**
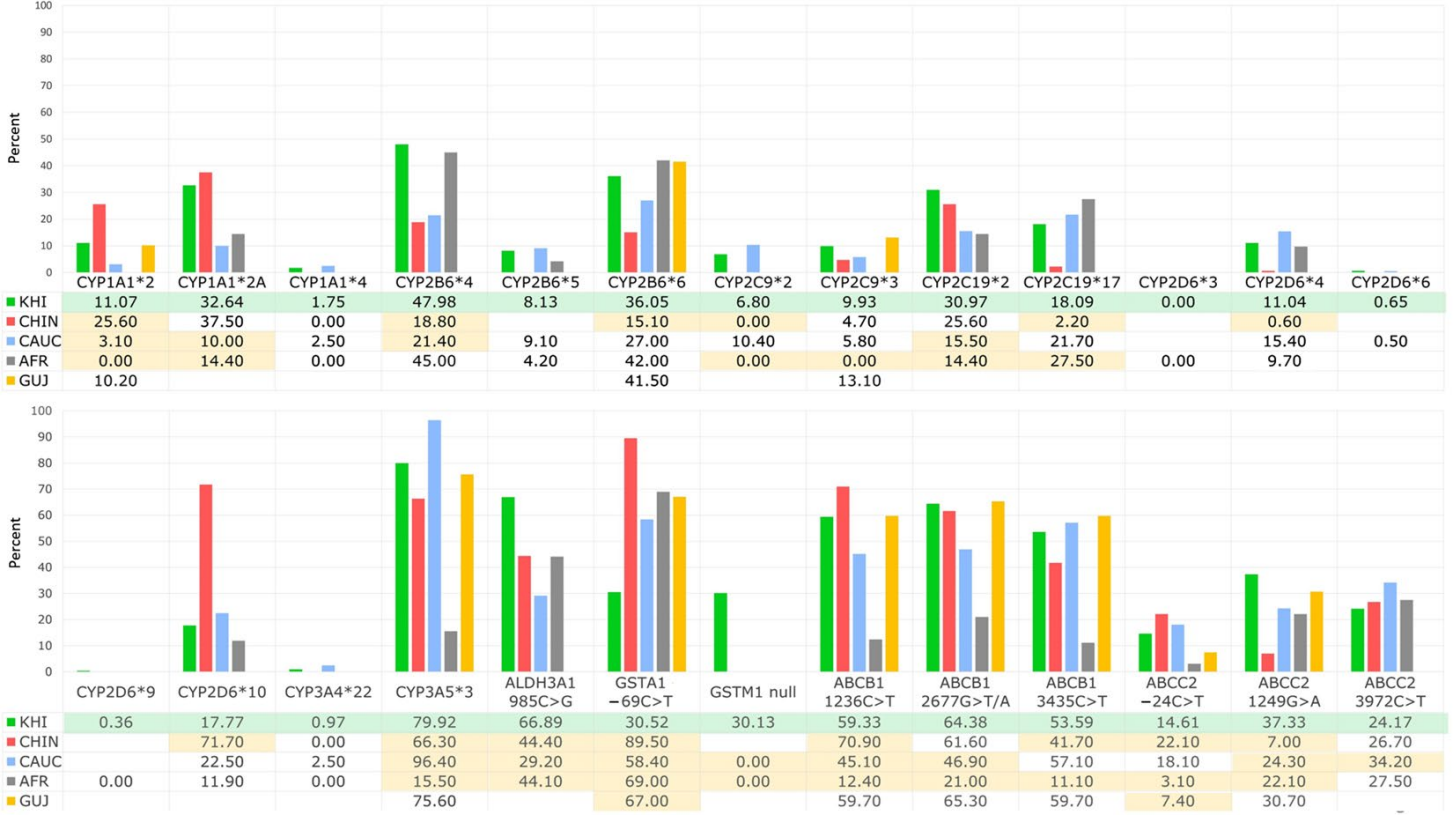
Variant allele frequencies (percent) of drug metabolizing enzymes and ABC transporters in healthy Pakistanis as compared to the HapMap Database (http://www.ncbi.nlm.nih.gov/SNP/). *KHI*, Karachi Pakistan (current study); *CHIN*, Chinese of Han ancestry; *CAUC*, Caucasians of Northern and Western European ancestry; *AFR*, African of Yoruba Nigerian ancestry; *GUJ*, Gujrati Indian ancestry living in Houston, Texas, USA. Green highlighted row shows current study sample and yellow shaded areas show significant difference from KHI samples computed through chi-square or Fisher exact test. The missing values indicate absence of data in HapMap database for that particular SNV.

Table 3 and Fig. 1 show variant allele frequencies. This study compared Pakistani population with major global ethnic groups (African of Yoruba Nigerian ancestry, Caucasian of Northern and Western European ancestry, Chinese of Han ancestry) as well as a subset from the neighbouring area of India (Gujrati Indians in Houston, Texas), all taken from the HapMap database. Variant allele frequencies were compared using Chi-square or Fischer exact tests. The results show that as compared to ethnicities in the HapMap database there were significant differences in prevalence of variant alleles of (a) *ALDH3A1*, (b) CYP1A1*2A, CYP2B6*4, CYP2B6*6, CYP2C19*2, CYP3A5*3, (c) *GSTA1*, and (d) *ABCB1* 2677G > T/A and *ABCC2* 1249G > A in our population. *GSTM1 null* genotype was found higher than in other reported ethnicities. CYP2D6*3 was absent in our study sample. All other SNVs showed intermediate or similar prevalence of variant alleles as compared to other ethnicities.

**Table 3:** Comparison of variant allele frequency with other ethnic groups. The Chi square value was computed with df = 1.

Genotype	KHI Sample	HapMap Ethnicities	Variant Allele %	*χ*² Value	p-value
CYP1A1*2	11.07	CHIN	25.6	7.05	0.008
		CAUC	3.1	4.83	0.028
		GUJ	10.2	0.04	0.84
		AFR	0	6.18	0.013
CYP1A1*2A	32.64	CHIN	37.5	0.52	0.47
		CAUC	10	15.28	<0.001
		GUJ	—	—	
		AFR	14.4	9.25	0.002
CYP1A1*4	1.75	CHIN	0	0.89	0.35
		CAUC	2.5	0.136	0.71
		GUJ	—	—	
		AFR	0	0.89	0.35
*CYP2B*6*4	47.98	CHIN	18.8	19.15	<0.001
		CAUC	21.4	15.6	<0.001
		GUJ	—	—	—
		AFR	45	0.18	0.67
CYP2B6*5	8.13	CHIN	—	—	
		CAUC	9.1	0.06	0.81
		GUJ	—	—	
		AFR	4.2	1.335	0.25
CYP2B6*6	36.05	CHIN	15.1	11.5	0.001
		CAUC	27	1.9	0.17
		GUJ	41.5	0.63	0.43
		AFR	42	0.74	0.39
*CYP2C*9*2	6.80	CHIN	0	3.6	0.056
		CAUC	10.4	0.82	0.36
		GUJ	—	—	
		AFR	0	3.6	0.056
CYP2C9*3	9.93	CHIN	4.7	2	0.16
		CAUC	5.8	1.18	0.28
		GUJ	13.1	0.49	0.48
		AFR	0	5.49	0.02
CYP2C19*2	30.97	CHIN	25.6	0.71	0.39
		CAUC	15.5	6.7	0.01
		GUJ	—	—	
		AFR	14.4	7.8	0.005
CYP2C19*17	18.09	CHIN	2.2	13.85	<0.001
		CAUC	21.7	0.41	0.52
		GUJ	-	-	
		AFR	27.5	2.5	0.11
CYP2D6*3	0.00	CHIN	—	—	
		CAUC	—	—	
		GUJ	—	—	
		AFR	0	0	1
CYP2D6*4	11.04	CHIN	0.6	9.94	0.002
		CAUC	15.4	0.83	0.36
		GUJ	—	—	
		AFR	9.7	0.1	0.75
CYP2D6*6	0.65	CHIN	—	—	
		CAUC	0.5	0.02	0.89
		GUJ	—	—	
		AFR	—	—	
CYP2D6*9	0.36	CHIN	—	—	
		CAUC	—	—	
		GUJ	—	—	
		AFR	0	0.18	0.67
CYP2D6*10	17.77	CHIN	71.7	58.8	<0.001
		CAUC	22.5	0.69	0.4
		GUJ	—	—	
		AFR	11.9	1.36	0.24
CYP3A4*22	0.97	CHIN	—	—	
		CAUC	2.5	0.69	0.4
		GUJ	—	—	
		AFR	—	—	
CYP3A5*3	79.92	CHIN	66.3	4.72	0.03
		CAUC	96.4	13	<0.001
		GUJ	75.6	0.54	0.46
		AFR	15.5	83.2	<0.001
*ALDH3A1 985C* > *G*	66.89	CHIN	44.4	10.25	0.001
		CAUC	29.2	28.46	<0.001
		GUJ	—	—	
		AFR	44.1	10.5	0.001
*GSTA1 -69C* > *T and -52G* > *A*	30.52	CHIN	89.5	72.48	<0.001
		CAUC	58.4	15.74	<0.001
		GUJ	67	26.63	<0.001
		AFR	69	29.62	<0.001
*GSTM1*	30.13	CHIN	—	—	
		CAUC	0	20.4	<0.001
		GUJ	—	—	
		AFR	0	20.4	<0.001
*ABCB1 1236C* > T or *8	59.33	CHIN	70.9	2.95	0.086
		CAUC	45.1	4.1	0.044
		GUJ	59.7	0.003	0.96
		AFR	12.4	47.88	<0.001
*ABCB1 2677G* > *T/A*	64.38	CHIN	61.6	0.17	0.68
		CAUC	46.9	6.19	0.01
		GUJ	65.3	0.02	0.89
		AFR	21	38.46	<0.001
*ABCB1 3435C* > *T*	53.59	CHIN	41.7	2.84	0.09
		CAUC	57.1	0.25	0.62
		GUJ	59.7	0.76	0.38
		AFR	11.1	41.26	<0.001
*ABCC2 -24C* > *T*	14.61	CHIN	22.1	1.87	0.17
		CAUC	18.1	0.44	0.5
		GUJ	7.4	2.65	0.1
		AFR	3.1	8.21	0.004
*ABCC2 3972C* > *T*	24.17	CHIN	26.7	0.17	0.68
		CAUC	34.2	2.43	0.12
		GUJ	—	—	
		AFR	27.5	0.29	0.6
*ABCC2 1249G* > *A*	37.33	CHIN	7	26.66	<0.001
		CAUC	24.3	3.98	0.046
		GUJ	30.7	0.98	0.322
		AFR	22.1	5.55	0.02

*KHI*, Karachi sample; *CHIN*, Chinese of Han ancestry; *CAUC*, Caucasian of Northern and Western European ancestry, *GUJ*, Gujrati Indians in Houston Texas; *AFR*, African of Yoruba Nigerian ancestry.

## Discussion

This study is the first comprehensive pharmacogenetic report from Pakistan. Previously we had shown that SNV prevalence of a select group of Phase-I as well as Phase-II drug metabolizing enzymes and ABC transporters in a breast cancer population sample had significant differences as compared to various ethnicities in HapMap database^19^. In this study also we identified several important differences between allele and genotype frequencies compared to other populations. Interestingly, the differences were similar to those reported previously for breast cancer population^19^, suggesting that real differences might exist. It is important to understand the implications of such differences in this population as compared to others as a first step to precision medicine globally. For example, an altered gene function can lead to unfavourable therapeutic outcome(s) in acute care or chronic management of various disorders. A few recent examples where gene variant necessitate adjusted dosing of a drug include that of clopidogrel in case of *CYP2C19* SNVs^21^, warfarin in case of *CYP2C9* or *VKORC1* SNVs^22^ and tamoxifen in case of *CYP2D6* SNVs^23^.

This study has many advantages. Pakistan is a populous multi-ethnic country with more than 200 million inhabitants. We included people from various major ethnic groups including Urdu-speaking, Balochi, Gujrati, Pashtun, Punjabi, Seraiki and Sindhi. The presence of a substantial proportion of Urdu-speaking population enabled us to extend the relevance of our results to neighbouring India which has a population over 1300 million. Overall their relevance could be extended to approximately 20–25% of the World’s population, which is historically underrepresented in pharmacogenomic studies. Hence, these results provide an important window to a largely unstudied population. Despite this, there are some limitations of our study. Approximately one third of our study population represented an inbred cohort due to consanguineous marriages, a widespread practice in this region. Further, our study cohort did not have substantial numbers of other Pakistani ethnic groups, like Baloch, Pashtun, Punjabi and Sindhi for sub-group analysis and robust conclusions regarding these ethnic groups. Hence, we recommend replicating the study to target these groups across the country and region.

The following section discusses in depth the implications of various significant observations in our study sample, comparing it with other ethnicities documented in the HapMap database, including African, Caucasian, Chinese and Gujrati. For further research and analysis, a web-based detailed account regarding substrates, inducers and inhibitors of various drug metabolizing enzymes, and updated clinical application of pharmacogenetics (CPIC guidelines) can be found at https://www.pharmgkb.org/, http://bioinformatics.charite.de/transformer/. and https://cpicpgx.org/guidelines/.

### CYP1A1

Cytochrome P450 1A1 metabolizes xenobiotics such as polycyclic aromatic hydrocarbons (PAHs) found in tobacco smoke, atmospheric pollutants and industrial waste and generates carcinogens from several substrates^24^. Hence, CYP1A1 is considered a link between environment-gene interaction in the etiology of various cancers such as head and neck cancers among smokers^25^. Our sample shows a higher prevalence of variant alleles (58% of diplotypes carried at least one variant allele) as compared to Caucasians and could potentially confer an elevated disease risk although this would need further validation. The difference in prevalence of SNVs could also have an impact on therapeutic outcome of drugs that may be favourable such as in case of antineoplastic docetaxel^26^, or first-line antiepileptics^27^, but unfavourable for antiemetic granisetron^28^.

### CYP2B6

Our results show a significantly higher prevalence of variant alleles CYP2B6*4 (48%) and CYP2B6*6 (36%) genotypes as compared to other ethnicities reported in HapMap database. Both alleles are associated with lower CYP2B6 activity leading to pharmacogenetic implications with many drugs including the antidepressant bupropion^29^, antiretroviral efavirenz^30^, anti-tuberculosis rifamycins and ethionamide^31^, among others. Pakistan has a high prevalence of tuberculosis, whereas HIV prevalence is on the rise especially in high-risk groups like sex workers and intravenous drug addicts^32^. Hence, compromised CYP2B6 function in the population could lead to elevated risk of side effects and drug-drug interactions. Further research is needed to evaluate the situation in this respect.

### CYP2C9

This enzyme metabolizes many drugs, such as warfarin^33^, phenytoin^34^ and non-steroidal anti-inflammatory drugs diclofenac and ibuprofen^35^. Our results show an intermediate prevalence of CYP2C9*2 genotype in KHI (6.8%) as compared to Caucasian (10.4%) and African (0%) populations in HapMap database. However, the CYP2C9*3 genotype is more frequent (9.93%) than in those population groups. Haplotype analysis suggests that approximately 30% population has some degree of compromised function of CYP2C9. The potential effects of this observation should be explored, especially for warfarin and phenytoin due to their narrow therapeutic index.

### CYP2C19

While CYP2C19*2 leads to complete loss-of-function, CYP2C19*17 is associated with gain-of-function. Several widely used drugs such as the antiplatelet clopidogrel^36^, antifungal voriconazole^37^, and the antidepressant citalopram^38^ are metabolized by CYP2C19. Because of problems in efficacy and pharmacokinetics, the USFDA and other such agencies include pharmacogenetic information in some drug labels to optimize the use of drugs, such as clopidogrel. Our data shows that only 27% of the Pakistani population had normal phenotype (CYP2C19*1/*1). Thus, further studies are required to elucidate the pharmacogenetics in this population, especially regarding drugs used in acute emergencies, such as clopidogrel in acute coronary syndrome.

### CYP2D6

This highly polymorphic enzyme is involved in the metabolism of more than 20% of drugs. Notable examples include the antidepressant paroxetine^38^, SERM tamoxifen^39^, antipsychotic clozapine^40^, adrenoceptor antagonists metoprolol and carvedilol among others^41^. Our data shows that the minor allele frequency was approximately 30%, whereas, 26% population had genotypes associated with some degree of functional loss.

### ALDH3A1

Aldehyde dehydrogenases are phase-1 metabolizing enzymes which exist as different isoenzymes. Our focus was ALDH3A1 which is involved in a broad spectrum of physiological activities, including the protection of oral and respiratory tract mucosa from damage caused by cigarette smoke^12^, food and air pollutants^42^, and ionizing radiation^43^. Additionally, it is involved in preventing ultraviolet light induced corneal damage^44^, detoxification of 4-HNE (4-hydroxynonenal; a by-product of lipid peroxidation)^9^, generation of NO from organic nitrates^36^, metabolism of oxazophorines like cyclophosphamide^8^, and synthesis of Coenzyme Q^11^. Thus, ALDH3A1 takes part in drug metabolism and reduction of oxidative stress. We had previously shown that the prevalence of *ALDH3A1* (985C > G) variant allele shows significant differences among various ethnicities in HapMap database and was much more prevalent (62.5%) in Pakistani breast cancer patients with 40% homozygous for variant allele^19^. In this study, we have shown that it is similarly prevalent in the healthy population (67% variant allele; 42% homozygous variant genotype). So far however, there is lack of concrete evidence that non-functional ALDH3A1 is associated with increased disease risk.

### GSTA1

Glutathione S-transferase A1 is the most abundant form of GSTs in human liver, kidney, adrenal gland and testis, where they appear to scavenge electrophiles and reduce oxidative stress^45^. It also appears to regulate other functions. For example, a recent *in vitro* study suggested that GSTA1 may facilitate nicotine-induced lung cancer metastasis^46^. Another study suggested its role in metabolism of anticancer drug busulfan^47^. We had previously reported that loss of GSTA1 is a major determinant of neutropenia among breast cancer patients receiving standard dose FAC (5-fluorouracil, doxorubicin, cyclophosphamide) chemotherapy^8^. Our current results also show that prevalence of variant allele is lower (30.5%) in the Pakistani population as compared to others in HapMap database (range: 58.4–89.5%) though in absolute terms it is still high.

### GSTM1

Glutathione S-transferase M1 is another GST believed to eliminate oxidative intermediates in the alimentary tract as posed by dietary toxins. The role of *GSTM1 null* genotype as a susceptibility factor for various carcinoma is conflicting, although a large meta-analysis comprising 198 studies revealed an association of lung cancer to *GSTM1 null* genotype^48^. Other studies have suggested that *GSTM1 null* genotype is associated with pathogenesis of chronic obstructive pulmonary disease^49^, or increased likelihood of toxicity of cyclophosphamide^50^ and oxaliplatin^51^. Our results show a high prevalence of putative “at risk” null genotype (59%). A recent study from Pakistan observed elevated levels of carcinogenic 1-hydroxypyrene in *GSTM1 null* carriers^52^, making further molecular epidemiological studies necessary in the Pakistani population.

### ABCB1

The ATP-binding cassette transporter B1, also called MDR1 (multi-drug resistance protein 1) or P-gp (permeability glycoprotein), is a membrane transporter located at many interfaces in the body^53^. It actively transports various xenobiotics and toxins across the cell membranes and has been implicated in antineoplastic drug resistance^54^. Certain drugs, such as amiodarone, clarithromycin, omeprazole, and calcium channel blockers, can inhibit this protein leading to drug-drug interactions^55^. Our results show a prevalence of 54–65% variant alleles of *ABCB1* (1236C > T, 2677G > T/A, 3435C > T; rs1128503, rs2032582, rs1045642 respectively). These frequencies are not significantly different from most populations except African.

### ABCC2

ATP-binding cassette transporter C2, also called MRP2 (multidrug resistance-associated protein 2) or CMOAT (Canalicular Multispecific Organic Anion Transporter), is an active efflux transporter identified at apical or biliary canalicular surfaces of hepatocytes and in the kidney. There is mounting evidence that by promoting efflux in target cells this protein is involved in the resistance to several drugs, such as antiepileptics^56^, antiretroviral drugs^57^, antineoplastic drugs^58^, and statins among others^59^. Conversely, its decreased function may lead to increased drug toxicity. Our data (Tables 2 and 3) suggests that a substantial proportion of the population has diplotypes with some degree of functional deficit where the prevalence of variant alleles ranges from 15–37%. Thus, the effects of this finding should be explored in terms of drug efficacy and toxicity.

In conclusion, this study showed that in our sample compared with other ethnic populations, there was a generally higher prevalence (p < 0.05) of variant alleles of *ALDH3A1*, CYP1A1*2A, CYP2B6*4, CYP2B6*6, CYP2C19*2, CYP3A5*3, ABCB1 2677G > T/A and *ABCC2* 1249G > A. Further, *GSTM1 null* genotype also had higher frequency. There is a lower prevalence of variant alleles of *GSTA1*, and *ABCC2* 3972C > T as compared to other ethnicities. As mentioned above, these results are not significantly different from our previously reported Pakistani female breast cancer patients^19^, thus suggesting real differences between our sample and other ethnicities in HapMap database, namely African, Caucasian, and Chinese. Hence, further research in other population cohorts within the country and the region would be beneficial for a more complete understanding of the pharmacogenetic landscape in a region which is underrepresented in genetic studies. This is an important step forward in achieving widespread and cost-effective implementation of personalized medicine in the community^60^.

## Methods

The study was conducted at Jinnah Medical and Dental College (JMDC), Karachi, Pakistan from July 2013 to December 2015 after approval by The Ethics Committee of Jinnah Medical & Dental College, in accordance with relevant guidelines. The study cohort included students and employees of JMDC who were invited to be volunteers and gave written informed consent.

Saliva was the source of genomic DNA. The saliva samples were collected and stored in Oragene® DNA collection kits (DNA Genotek Inc. Canada) according to manufacturer’s recommendations. DNA was extracted through the proprietary extraction kit provided with collection kits. The extracted DNA was air-shipped to the Institute of Experimental and Clinical Pharmacology, Christian-Albrechts University, Kiel, Germany for genotyping.

For primers and experimental method details, see Supplementary Tables 1 and 2. Briefly, genotyping was performed by restriction fragment length polymorphism (RFLP) for *CYP1A1* SNVs rs1048943 (g.3798C > T, *2A and *2B), rs1799814 (g.2454A > G, *2B) and rs4646903 (g.2452C > A, *4); for *CYP2B6* SNVs rs2279343 (c785A > G, *4), rs3211371 (c.1459C > T, *5) and rs3745274 (c.516G > T, *6); for *CYP2C9* SNVs rs1799853 (430C > T, *2) and rs1057910 (1075A > C, *3); for CYP2C19*2 SNV rs4244285 (681G > A); for *CYP2D6* SNVs rs5030655 (g.1707delT, *6), rs3892097 (g.1846C> T, *4), rs35742686 (g.2549delA, *3), rs5030656 (g.2615-g.2617delAAG, *9 and rs1065852 (g.100C > T), *10); and for *CYP3A5* SNV rs776746 (g.6986G > A, *3). The *CYP2D6* deletion (*5) and gene duplication could not be determined in the DNA retrieved from saliva specimen. All homozygous variant genotypes detected through RFLP were repeated to ensure accuracy.

A PSQ HS 96 (Qiagen, Hilden, Germany) was used for pyrosequencing (PSQ) SNVs for *GSTA1* −69C > T and −52G > A (rs3957357 and rs3957356 respectively; representing GSTA1*A and GSTA1*B haplotype); for *ALDH3A1* (985C > G; rs2228100), and for *CYP3A4* (15389C > T; rs35599367. Novel PSQ methods were established for CYP2C19*17, ABCB1 SNVs (rs1128503, g.1236C > T; rs2032582 g.2677G > T/A; rs1045642 g.3435C > T), and for ABCC2 (rs717620 −g.24C > T, rs2273697 g.1249G > A, rs3740066 g.3972C > T). For all the PCR reactions, a GeneAmp PCR 9700 Thermocycler (Applied Biosystems, Darmstadt, Germany) was used.

The data was analysed using SPSS® version 19.0 software (IBM, Ehningen, Germany). The results were entered as frequencies, and percentage and 95% confidence interval (proportions) was calculated. All genotype frequencies were tested and found to be within Hardy-Weinberg equilibrium. Allele frequency data was compared through χ^2^ or Fisher’s Exact test where applicable. A p-value < 0.05 was considered significant.

## Supplementary information

Supplementary Tables 1 & 2

## Data Availability

The authors undertake that materials, data and associated protocols would be made available to readers.
