# Supplementary material for: Implications of genetic variation of common Drug Metabolizing Enzymes and ABC Transporters among the Pakistani Population
Source: Sci Rep. 2019 May 13;9:7323. doi: 10.1038/s41598-019-43736-z (PMC6514210; doi:10.1038/s41598-019-43736-z)
Supplement: Supplementary file 1 — Supplementary Tables 1 & 2 [file 41598_2019_43736_MOESM1_ESM.doc]

**Implications of genetic variation of common Drug Metabolizing Enzymes and ABC Drug Transporters among the Pakistani Population**

**Running Title:** DMET SNVs in Pakistani population

**Authors:**

^1^Nasir Ali Afsar*, ^2^Henrike Bruckmueller, ^2,3^Anneke Nina Werk, ^1^Muhammad Kashif Nisar, ^4^HR Ahmad, ^2^Ingolf Cascorbi.

^1^Jinnah Medical and Dental College, Sohail University, 22-23 Shaheed-e-Millat Road, Karachi-75400, Pakistan.

^2^Institute of Experimental and Clinical Pharmacology, Christian Albrechts University Kiel, Hospitalstr. 4, Kiel-24105, Germany.

^3^Department of Internal Medicine I, University Medical Center Hamburg-Eppendorf, Hamburg, Germany.

^4^Department of Biological and Biomedical Sciences, The Aga Khan University, Karachi, Pakistan. Sindh Institute of Urology and Transplantation, Karachi, Pakistan.

MKN is currently at Liaquat National Hospital & Medical College, Karachi, Pakistan.

**All correspondence to:**

Nasir Ali Afsar, Department of Pharmacology, Jinnah Medical and Dental College, Sohail University, 22-23 Shaheed-e-Millat Road, Karachi-75400, Pakistan. Phone: +92-333-3499670, e-mail: [drnasirpk@yahoo.com](mailto:drnasirpk@yahoo.com)

**Supplementary Table 1. Primers used to determine genetic variants in selected drug metabolizing enzymes and transporters the Pakistani population.** (Implications of genetic variation of common Drug Metabolizing Enzymes and ABC Drug Transporters among Pakistani Population).

| **Gene** | **rs number** | **SNV** | **Forward Primer (5’ to 3’)** | **Reverse Primer (5’ to 3’)** | **Sequencing Primer (5’ to 3’)** | **Method** |
| --- | --- | --- | --- | --- | --- | --- |
| *CYP1A1* | rs1799814 | g.2452C>A | TTC CAC CCG TTG CAG CAG GAT AGC C | CTG TCT CCC TCT GGT TAC AGG AAG | None | RFLP^1^ |
|  | rs1048943 | g.2454A>G | TTC CAC CCG TTG CAG CAG GAT AGC C | CTG TCT CCC TCT GGT TAC AGG AAG | None | RFLP^1^ |
|  | rs4646903 | g.3798T>C | GGC CCC AAC TAC TCA GAG GCT | GGC TGA GCA ATC TGA CCC TA | None | RFLP^1^ |
| *CYP2B6* | rs3745274 | c.516G>T | GGT CTG CCC ATC TAT AAA C | CTG ATT CTT CAC ATG TCT GCG | None | RFLP^2^ |
|  | rs2279343 | c.785A>G | TAA TTT TCG ATA ATC TCA CTC CTG C | CTC CCT CTG TCT TTC ATT CTG T | None | RFLP^2^ |
|  | rs3211371 | c.1459C>T | TGA GAA TCA GTG GAA GCC ATA GA- | TAA TTT TCG ATA ATC TCA CTC CTG C | None | RFLP^2^ |
| *CYP2C9* | rs1799853 | c.430C>T | TAC AAA TAC AAT gAA AAT ATC ATg | CTA ACA ACC AgA CTC ATA ATg | None | RFLP^3^ |
|  | rs1057910 | c.1075A>C | AAT AAT AAT ATg CAC gAg gTC CAg AgA TgC | gAT ACT ATg AAT TTg ggA CTT C | None | RFLP^3^ |
| *CYP2C19* | rs4244285 | c.681G>A | AAT TAC AAC CAg AgC TTg gC | TAT CAC TTT CCA TAA AAg CAA g | None | RFLP^4^ |
|  | rs12248560 |  | TGA TGG AGA AGG GAG AAC TCT TA | Bio-TCG TGG CGC ATT ATC TCT TA | TTG TGT CTT CTG TTC TCA A | PSQ* |
| *CYP2D6* | rs1065852 | g.100C>T | GCC GTG ATA GTG GCC ATC T | Bio-ACC TGG TCG AAG CAG TAT GG | CTG GGC TGC ACG CTA | RFLP* |
|  | rs5030655 | g.1707delT | GAG ACT CCT CGG TCT CTC G | CCT GGG CAA GAA GTC GCT GA CCA G |  | RFLP^5^ |
|  | rs3892097 | g.1846C>T | GAG ACT CCT CGG TCT CTC G | CCT GGG CAA GAA GTC GCT GA CCAG |  | RFLP^5^ |
|  | rs35742686 | g.2549delA | GCT GGG GCC TGA GAC TT | GGC TGG GTC CCA GGT CAT AC |  | RFLP^5^ |
|  | rs5030656 | g.2615-g.2617delAAG | AGG CCT TCC TGGCAG AGA TGA AG | CCC CTG CAC TGT TTC CCA GA |  | RFLP^5^ |
| *CYP3A4* | rs35599367 | g.15389C>T | GTC TTC CTA TGA TGG GCT CCT TG | Bio GTT ATC AGG TGC CAG TGA TGC | TCT CCA TCA CAC CCA | PSQ^6^ |
| *CYP3A5* | rs776746 | g.6986G>A | CTT GCA GCA TTT AGT CCT TG | GGT CCA AAC AGG GAA GAG GT | None | RFLP^7^ |
| *ALDH3A1* | rs2228100 | c.985G>C | Bio-GGG GTC TAG GTG CTT GCA CT | ACA GGC CCG AAG ATC TCC | CCT CTT GCA TCA CCG | PSQ^8^ |
| *GSTA1* | rs3957357 | g.-69C>T | GTT AAA CGC TGT CAC CGT CCT G | Bio-GGA GTG GCT TTT CCC TAA CTT GA | CTC CCA CTG AAA GAA G | PSQ^8^ |
|  | rs3957356 | g.-52G>A | GTT AAA CGC TGT CAC CGT CCT G | Bio-TGG GAG TGG CTT TTC CCT AAC TT | TTT GTT CCT CTC AAT AGT TC | PSQ^8^ |
| *GSTM1* | *Deletion* | **1 / *0* | CGC CAT CTT GTG CTA CAT TGC CCG | TTC TGG ATT GTA GCA GAT CA | None | PCR^9^ |
| *ABCB1* | rs1128503 | g.1236C>T | TCC TGT GTC TGT GSS TTG CCT | Bio-GTC TAG CTC GCA TGG GTC AT | TGG TAG ATC TTG AAG GG | PSQ* |
|  | rs2032582 | g.2677G>T/A | Bio-gCA ggA gTT gTT gAA ATg AAA AT | AAT ggC CTg AAA ACT gAA AAA gTC | TTA gTT TgA CTC ACC TTC C | PSQ* |
|  | rs1045642 | g.3435 C>T | Bio-GGA GCC CAT CCT GTT TGA C | TAG GCA GTG ACT CGA TGA AGG | CTC CTT TGC TGC CCT | PSQ* |
| *ABCC2* | rs717620 | g.-24C>T | ggT CAT CCT TTA Cgg AgA ACA T | Bio-gCA gAA CTT CTC CAg CAT gAT T | TCA TAT TAA TAg AAg AgT CT | PSQ* |
|  | rs2273697 | g. 1249G>A, | Bio-CAA CTT ggC CAg gAA ggA gTA | TTC Tgg gCA TCC ACA gAC A | TCA ggT TCA CTg TTT CTC | PSQ* |
|  | rs3740066 | g.3972C>T | Bio-CTG GTC CTC AGA GGG ATC ACT T | TCC ACC TAC CTT CTC CAT GCT AC | CCT ACC TTC TCC ATG CTA | PSQ* |

*RFLP*, Restriction Fragment Length Polymorphism; *PSQ*, Pyrosequencing; *Novel method (See Supplementary Table 2)

**References – Supplementary Table 1:**

1. Cascorbi, I., Brockmöller, J., & Roots, I. A C4887A polymorphism in exon 7 of human CYP1A1: population frequency, mutation linkages, and impact on lung cancer susceptibility. *Cancer Res*. **56**, 4965-4969 (1996).
2. Lang, T. *et al*. Extensive genetic polymorphism in the human CYP2B6 gene with impact on expression and function in human liver. *Pharmacogenetics*. **11**, 399–415 (2001).
3. Sullivan-Klose, T.H. *et al*. The role of the CYP2C9-Leu359 allelic variant in the tolbutamide polymorphism. *Pharmacogenetics*. **6**, 341–349 (1996).
4. de Morais, S.M. *et al*. The major genetic defect responsible for the polymorphism of S-mephenytoin metabolism in humans. *J. Biol. Chem*. **269**, 15419–15422 (1994).
5. Sachse, C., Brockmöller, J., Bauer, S., & Roots, I. Cytochrome P450 2D6 variants in a Caucasian population: allele frequencies and phenotypic consequences. [*Am. J. Hum. Genet.*](https://www.ncbi.nlm.nih.gov/pubmed/?term=sachse-c+1997) **60**, 284-295 (1997).
6. Bruckmueller, H. *et al*. Which Genetic Determinants Should be Considered for Tacrolimus Dose Optimization in Kidney Transplantation? A Combined Analysis of Genes Affecting the CYP3A Locus. *Ther. Drug Monit*. **37**, 288-295 (2015).
7. Hustert, E. *et al*. The genetic determinants of the CYP3A5 polymorphism. *Pharmacogenetics*. **11**, 773–779 (2001).
8. Afsar, N.A. *et al*. Genotype Frequencies of selected Drug Metabolizing Enzymes and ABC Drug Transporters among Breast Cancer Patients on FAC Chemotherapy. *Basic Clin. Pharmacol. Toxicol*. **107**, 570-576 (2010).
9. Brockmöller, J., Kerb, R., Drakoulis, N., Staffeldt, B., & Roots, I. Glutathione S-transferase Ml and its variants A and B as host factors of bladder cancer susceptibility: a case-control study. *Cancer Res*. **54**, 4103-4111 (1994).

**Supplementary Table 2. Novel methods to genotype drug metabolizing enzymes and ABC transporters.** The PCR thermocycler conditions are given below in the table. The PSQ conditions are similar to those mentioned previously^1^. (Implications of genetic variation of common Drug Metabolizing Enzymes and ABC Drug Transporters among Pakistani Population).

| **Gene** | **rs number** | **SNV** | **Tech-nique** | **Initial Hold** | **Amplification cycle** | **Post Hold** | **Cycles** | **Amplicon (bp)** |
| --- | --- | --- | --- | --- | --- | --- | --- | --- |
| *CYP2D6* | rs1065852 | g.100C>T | RFLP | 94^o^C, 5 min | 94^o^C, 30 sec; 56^o^C, 20 sec; 72^o^C, 20 sec | 72^o^C, 5 min | 30* | 325 |
| *CYP2C19* | rs12248560 | g.-806C>T | PSQ | 95oC, 5 min | 95oC, 30 sec; 59oC, 30 sec; 72oC, 30 sec | 72^o^C, 7 min | 45 | 251 |
| *ABCB1* | rs1128503 | g.1236C>T | psq | 95^o^C, 5 min | 95^o^C, 30 sec; 52^o^C, 60 sec; 72^o^C, 30 sec | 72^o^C, 7 min | 50** | 201 |
|  | rs2032582 | g.2677G>T/A | psq | 94^o^C, 4 min | 94^o^C, 30 sec; 65^o^C, 30 sec; 72^o^C, 30 sec | 72^o^C, 7 min | 45 | 311 |
|  | rs1045642 | g.3435 C>T | psq | 94^o^C, 4 min | 94^o^C, 30 sec; 63^o^C, 30 sec; 72^o^C, 30 sec | 72^o^C, 7 min | 45 | 134 |
| *ABCC2* | rs717620 | g.-24C>T | psq | 94^o^C, 4 min | 94^o^C, 30 sec; 63^o^C, 30 sec; 72^o^C, 30 sec | 72^o^C, 7 min | 45 | 149 |
|  | rs2273697 | g.1249G>A, | psq | 94^o^C, 4 min | 94^o^C, 30 sec; 63^o^C, 30 sec; 72^o^C, 30 sec | 72^o^C, 7 min | 45 | 66 |
|  | rs3740066 | g.3972C>T | psq | 94^o^C, 4 min | 94^o^C, 30 sec; 61^o^C, 30 sec; 72^o^C, 30 sec | 72^o^C, 7 min | 45 | 54 |

*RFLP*, restriction fragment length polymorphism; *PSQ*, pyrosequencing.

Except stated specifically, all reactions included, 2.5 μL 10x buffer, 1.25 μL MgCl_2_ 50mM, 2.5 μL dNTPs 2.5mM, 0.15 μL Taq polymerase, 0.5 μL forward and backward primers each, PCR water and gDNA to make 25 μL PCR reaction volume.

*DMSO 1 μL, dNTPs 1 μL, 0.25 μL Taq polymerase; all other constituents similar to other SNVs. Restriction enzyme *Hph*-I; incubation at 37°C for 6 hours and 65°C for 20 minutes. Electrophoresis bands at 282 and 62 bp in wild-type; mutation yields additional bands at 183 and 100 bp.

**1.0 μL MgCl_2_ 50mM; all other constituents similar to other SNVs.

**References – Supplementary Table 2:**

1. Afsar, N.A. *et al*. Genotype Frequencies of selected Drug Metabolizing Enzymes and ABC Drug Transporters among Breast Cancer Patients on FAC Chemotherapy. *Basic Clin. Pharmacol. Toxicol*. **107**, 570-576 (2010).
